# On the Rarefied Gas Experiments

**DOI:** 10.3390/e21070718

**Published:** 2019-07-23

**Authors:** Róbert Kovács

**Affiliations:** 1 Department of Energy Engineering, Faculty of Mechanical Engineering, Budapest University of Technology and Economics (BME), 1111 Budapest, Hungary; kovacs.robert@wigner.mta.hu; 2 Department of Theoretical Physics, Wigner Research Centre for Physics, Institute for Particle and Nuclear Physics, 1121 Budapest, Hungary; 3 Montavid Thermodynamic Research Group, 1112 Budapest, Hungary

**Keywords:** rarefied gases, non-equilibrium thermodynamics, kinetic theory

## Abstract

There are limits of validity of classical constitutive laws such as Fourier and Navier-Stokes equations. Phenomena beyond those limits have been experimentally found many decades ago. However, it is still not clear what theory would be appropriate to model different non-classical phenomena under different conditions considering either the low-temperature or composite material structure. In this paper, a modeling problem of rarefied gases is addressed. The discussion covers the mass density dependence of material parameters, the scaling properties of different theories and aspects of how to model an experiment. In the following, two frameworks and their properties are presented. One of them is the kinetic theory based Rational Extended Thermodynamics; the other one is the non-equilibrium thermodynamics with internal variables and current multipliers. In order to compare these theories, an experiment on sound speed in rarefied gases at high frequencies, performed by Rhodes, is analyzed in detail. It is shown that the density dependence of material parameters could have a severe impact on modeling capabilities and influences the scaling properties.

## 1. Introduction

The classical material laws such as Fourier and Navier-Stokes are acceptable for tasks concerning homogeneous materials, dense gases, and far from low-temperatures (20 K). In the engineering practice, these constitutive equations are well-known and widely used. Nevertheless, there are situations where some generalizations must be applied. Such a case could occur on small (micro or nano) length scales, short time scales, near low-temperature or far from equilibrium.

It is easier to understand the origin of the present continuum model of non-equilibrium thermodynamics with internal variables (NET-IV) for rarefied gases together with its properties if a purely heat conduction problem is presented first, since the essential attributes are inherited. Moreover, the most visible differences are analogous in both cases, for instance, the available degrees of freedom, the structure of the equations and the interpretation of the parameters. All these differences originate at the roots of these approaches.

Considering only heat conduction, various forms of heat propagation are experimentally found [1,2,3,4,5,6,7,8,9]. These are called second sound and ballistic propagation [9,10,11,12]. Their modeling background is diverse, and one can find many extended heat conduction equations with many different interpretations in the literature [13,14,15,16,17,18,19,20]. One of them is related to the approach of Rational Extended Thermodynamics (RET) [9,21], it considers kinetic theory rigorously and uses phonon hydrodynamics to describe these deviations from Fourier’s law [22,23]. Another approach uses non-equilibrium thermodynamics with internal variables and current multipliers (NET-IV) [12]. Both are tested on the same heat conduction experiment, and the latter one seems to be more effective [23].

The main difference between these two approaches is routed to the physics that lies behind the system of constitutive equations. Using kinetic theory, one always has to assume apriori a mechanism occurring between the particles and describe the interaction among them. On the other side, non-equilibrium thermodynamics is phenomenological, the derivation of constitutive equations does not require any assumption regarding the microstructure, which makes the model more general and, in parallel, offers more degrees of freedom by not restricting the coupling coefficients. In the kinetic theory, due to the prescribed interaction model, most of the coefficients can be calculated, and only a few of them have to be fitted to the experimental data. Although its mathematical structure is advantageous, it is symmetric hyperbolic [21,24], the fixed parameters lead to its weakness: e.g., in a previously mentioned heat conduction problem, one has to use at least 30 momentum equations with increasing tensorial order to obtain the ballistic propagation speed approximately. The approach of NET-IV can resolve this problem, also preserving the structure of momentum equation; however, in order to fit, it requires more parameters [12,22,23,25]. All these approaches have advantages and disadvantages, and their detailed comparison is presented in [26].

In the case of investigating room temperature non-Fourier phenomenon, the phonon picture is not applicable [25]. One advantage of NET-IV is that it is applicable and tested on room temperature experiments that show over-diffusive type non-Fourier heat propagation [27,28,29]. It makes the kinetic approach of heat conduction more challenging to apply for practical problems; however, there are situations where its predictive power is useful (e.g., estimating transport coefficients). Such a situation is related to the topic of rarefied gases [30]. In some senses, the behavior of a rarefied gas (i.e., a gas under low pressure) is analogous with a rarefied phonon gas that applied in case of heat conduction. The difference among them is the type of the particle and the interpretation of some physical quantities. In order to understand the analogy, the ballistic conduction must be defined.

Using phonon hydrodynamics, the ballistic heat conduction is interpreted as non-interacting particles that scatter on the boundary only, i.e., traveling through the material without any collision [9]. It is important to emphasize that the following Equations (1) and (2) describe not merely a ballistic phenomenon but together with the diffusion and second sound propagation modes. This assumption leads to the system of equations in one spatial dimension:

(1) $$\begin{array}{cc} \partial_{t}e+c^{2}\partial_{x}p= & 0,\\ \partial_{t}p+\frac{1}{3}\partial_{x}e+\partial_{x}N= & -\frac{1}{\tau_{R}}p,\\ \partial_{t}N+\frac{4}{15}c^{2}\partial_{x}p= & -\left(\frac{1}{\tau_{R}}+\frac{1}{\tau_{N}}\right)N, \end{array}$$

where *e* being the energy density, *p* is momentum density, *c* stands for the Debye speed, $\tau_{R}$ and $\tau_{N}$ are the relaxation times referring to the resistive and normal processes [9], furthermore, $\partial_{t}$ denotes the partial time derivative, applied for a rigid heat conductor. Here, *N* is the deviatoric part of the pressure tensor. In phonon hydrodynamics, it can be identified as a current density of the heat flux. The key aspect to include ballistic contributions into the modeling is achieving coupling between the heat flux and the pressure. This is one merit of this approach: such coupling was not realized in any other theories before. That was the motivation for the approach of NET-IV, this coupling is obtained using current multipliers [31], and the same structure can be reproduced [12,25]:

(2) $$\begin{array}{cc} \rho c\partial_{t}T+\partial_{x}q= & 0,\\ \tau_{q}\partial_{t}q+q+\lambda \partial_{x}T+\kappa \partial_{x}Q= & 0,\\ \tau_{Q}\partial_{t}Q+Q+\kappa \partial_{x}q= & 0, \end{array}$$

where *Q* plays the role of *N*, *q* is the heat flux, *c* denotes the specific heat and the coefficient κ is not fixed on contrary to (1), this property allows to adjust the exact propagation speed using only 3 equations instead of 30. The properties of the models above are discussed deeply by Jou et al. [32], Alvarez et al. [33] and Guo et al. [34].

Despite the numerous differences between phonons and real molecules, the situation is similar for rarefied gases, at least at the level of entropy; Equation (3) does not contain restrictions about the type of the fluid, i.e., there is an “entropic equivalence” between them. Here, a gas under low pressure consists few enough particles to observe the ballistic contribution. In NET-IV, the starting point is the generalization of entropy density and its current:

(3) $$\begin{array}{cc} s(e,\rho ,q_{i},\Pi_{ij}) & =s_{e}\left(e,\rho \right)-\frac{m_{1}}{2}q_{i}q_{i}-\frac{m_{2}}{2}\Pi_{\langle ij\rangle }\Pi_{\langle ij\rangle }-\frac{m_{3}}{6}\Pi_{ii}\Pi_{jj},\\ J_{i} & =\left(b_{\langle ij\rangle }+b_{kk}\delta_{ij}/3\right)q_{j}, \end{array}$$

then exploiting the entropy production inequality of second law [12], one obtains a continuum model compatible with the kinetic theory to model rarefied gases [25,26,35]. Einstein’s summation convention is applied. Here $\Pi_{ij}$ is an internal variable [36,37,38], it is identified as the viscous pressure, $\Pi_{ij}=P_{ij}-p\delta_{ij}$ with *p* being the hydrostatic pressure, in accordance with EIT [18,19]. This is the usual assumption in theories of Extended Thermodynamics, as a consequence of the compatibility with kinetic theory [21,32], it also includes Meixner’s theory [39], the first extension of Navier-Stokes equation. Besides, $b_{ij}$ is called Nyíri-multiplier (or current multiplier) [31] which permits obtaining coupling between the heat flux and the pressure. Furthermore, the form of entropy flux (3) is compatible with the one proposed by Sellitto et al. [40], where the gradient of heat flux acts as a multiplier. Since the proper description requires the separation of deviatoric and spherical parts, in Equation (3) 〈〉 denotes the traceless part of the pressure. Equation (3) presents the same generalization as used for modeling complex heat conduction processes that include diffusive and wave propagation modes together, thus, hereinafter it is called *non-local generalization* of entropy and its current density [25]. The linearized-generalized Navier-Stokes-Fourier system reads in one dimension [25]:

(4) $$\begin{array}{cc} \tau_{q}\partial_{t}q+q+\lambda \partial_{x}T-\alpha_{21}\partial_{x}\Pi_{s}-\beta_{21}\partial_{x}\Pi_{d}= & 0,\\ \tau_{d}\partial_{t}\Pi_{d}+\Pi_{d}+\nu \partial_{x}v+\beta_{12}\partial_{x}q= & 0,\\ \tau_{s}\partial_{t}\Pi_{s}+\Pi_{s}+\eta \partial_{x}v+\alpha_{12}\partial_{x}q= & 0, \end{array}$$

where the lower indices *d* and *s* denote the deviatoric and spherical parts, respectively. The η is the bulk viscosity, ν denotes the shear viscosity, $\alpha_{ab}$, $\beta_{ab}$ (a,b=1,2) are the coupling parameters between the heat flux and the pressure and $\tau_{m}$ (m=q,d,s) are the relaxation times, here the coupling parameters and the relaxation times are to be fit. This structure is equivalent to the 1D linearized version model from RET [41,42,43]:

(5) $$\begin{array}{cc} \tau_{q}\partial_{t}q+q+\lambda \partial_{x}T-RT_{0}\tau_{q}\partial_{x}\Pi_{d}+RT_{0}\tau_{q}\partial_{x}\Pi_{s}= & 0,\\ \tau_{d}\partial_{t}\Pi_{d}+\Pi_{d}+2\nu \partial_{x}v-\frac{2\tau_{d}}{1+c_{v}^{*}}\partial_{x}q= & 0,\\ \tau_{s}\partial_{t}\Pi_{s}+\Pi_{s}+\eta \partial_{x}v+\frac{\tau_{s}\left(2c_{v}^{*}-3\right)}{3c_{v}^{*}\left(1+c_{v}^{*}\right)}\partial_{x}q= & 0, \end{array}$$

with *R* being the gas constant and $c_{v}^{*}$ denotes the dimensionless specific heat: $c_{v}^{*}=c_{v}/R$. As it is apparent, only the relaxation times are free parameters, all the other coefficients are fixed. It is interesting to note that the system (5) is derived by considering a doubled hierarchy of balance equations [24,44]. The reason behind that fact is related to the more degrees of freedom within polyatomic gases [26]. It is also important to note that it is not the only way for the kinetic theory: Lebon and Cloot derived a possible generalization using gradient terms as new variables [45] to model nonlocal phenomena. In order to obtain a complete (closed) system of equations, beside the constitutive equations above, one has to use the balance laws as well:

(6) $$\begin{array}{cc} \partial_{t}\rho +\rho_{0}\partial_{x}v= & 0,\\ \rho_{0}\partial_{t}v+\partial_{x}\Pi_{d}+\partial_{x}\Pi_{s}+RT_{0}\partial_{x}\rho +R\rho_{0}\partial_{x}T= & 0,\\ \rho_{0}c\partial_{t}T+\partial_{x}q+R\rho_{0}T_{0}\partial_{x}v= & 0, \end{array}$$

i.e., the mass, momentum and energy balances, respectively.

It is worth mentioning the earlier works of Lebon and Cloot [45] and Carrassi and Morro [46,47] where a similar comparison is performed. In these papers, other experiments are analyzed that are conducted by Meyer and Sessler [48], which slightly differ from the following one.

Before discussing the experimental observations about rarefied gases, one must define what a rarefied gas is. According to Klimontovich, a density parameter ε should be small for rarefied gases: ε<<1. It expresses the ratio of the occupied volume by the molecules and the overall available volume. Using the air properties at atmospheric pressure and room temperature, it turns out that $\varepsilon \approx 10^{-4}$ [49]. In other words, air at 10 atm is also rarefied or at least close to a rarefied state. Eventually this definition is not appropriate as it takes the volume corrections only into account, leaving the Knudsen number out of sight. The validity limit of the classical transport equations can be given more appropriately using the Knudsen number since it includes the mean free path as a characteristic quantity of a process. Above Kn≈0.05−0.1 the generalizations of the Navier-Stokes-Fourier equations must be applied [30,35].

In the following, the particular experimental observations of Rhodes are presented. That experiment highlights two essential aspects which are not independent of each other, namely, the density dependence of material parameters and the frequency/pressure (ω/p) scaling properties of the RET and NET-IV models. The discussion aims to present the necessary requirements of obtaining ω/p-dependence from continuum point of view. At the end, that measurement is evaluated using the framework of NET-IV and compared to the approach of RET.

## 2. Experiments

As in the case of heat conduction [23], the experimental results are considered as a benchmark problem in order to test the validity and feasibility of the corresponding generalized model. Here, one measurement performed by Rhodes [50] is discussed in detail. There are many other data in the literature [51,52,53,54], but this one is going to be sufficient to present all the necessary conclusions and difficulties arising in that field, i.e., how the scaling properties appear, the interpretation of the experiments and more importantly, the role of the material parameters.

**Figure 1:**
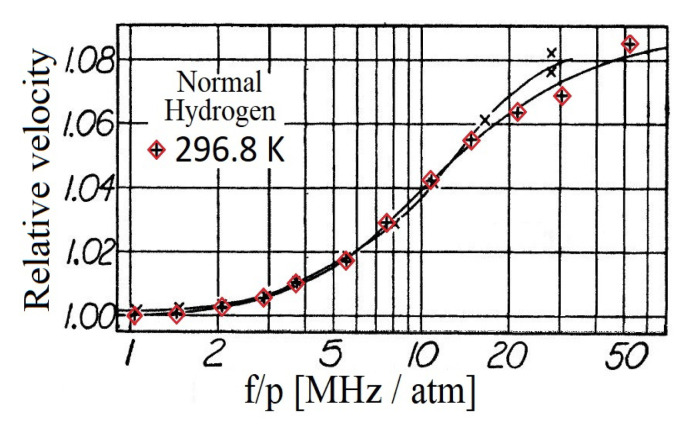
Speed of sound measurement performed by Rhodes [50]. The vertical axis denotes the relative speed of sound, i.e., $v/v_{0}$, where $v_{0}$ is the speed of sound related to the normal state. The original data can be found in [50]. The relevant points are emphasized by red squares.

A sonic interferometer [55] is used to measure the sound speed for various frequency-pressure ratios [50], see Figure 1 for typical data. The interferometer is placed in a dewar to maintain a constant temperature within.

It has to be emphasized that the frequency was constant as well in the experiments [50], i.e., the pressure is varied over the whole interval. More appropriately, it was the density that changed during the experiment when the constant temperature has maintained. In Figure 1, the results related to normal Hydrogen is presented. The measurement is also performed using pure para-Hydrogen and the 50–50 mixture of para-ortho Hydrogen [50]. Now choosing the curve from Figure 1 corresponding to 296.8 K. Before making any advancement with the extended models, two essential aspects must be discussed. The first one is to investigate the density dependence of material parameters. Then, one can calculate the dispersion relation for the relating model (4)–(6) or (5) and (6) to model the experiment and analyze the frequency-pressure dependency, too.

### 2.1. Density Dependence of Material Parameters

Despite the fact that the experimental data are recorded as a function of ω/p only, it is also emphasized in the related paper of Rhodes [50] that the frequency was constant all along the measurement in an isothermal environment. It means that the pressure is varied by changing the density of the gas only. That is, changing the state of the gas from normal (or dense) state to a rarefied one must reflect the role of density dependence of material parameters.

Indeed, this is an efficient way to demonstrate the validity region of the classical Navier-Stokes-Fourier equations. As the experiment shows, some effects become essential when the gas reaches its rarefied state. Density-dependent material coefficients could represent it: some of them make the related memory and nonlocal effects (couplings) negligible for dense states and worthwhile to account in rarefied states while the others change accordingly. This is a natural expectation from theoretical point of view.

Considering the RET approach of Arima et al. [43], one can notice the following:

- the viscosities are constant: $\nu =p_{0}\tau_{d}$ for shear viscosity and $\eta ∼p_{0}\tau_{s}$ for the bulk part,
- the thermal conductivity is also constant: $\lambda ∼cp_{0}\tau_{q}$ (in which *c* being the specific heat),
- as a consequence of the above, the relaxation times are inversely proportional with mass density: $\tau ∼\frac{1}{\rho }$.

All the other parameters are fixed and do not depend on the mass density in any way. It is important to emphasize that the essential material parameters used in their analysis are constant for very different pressures, like $10^{3}$ or $10^{5}$ Pa. These viscosities are derived on kinetic theory considerations (see, e.g., [56,57,58]) and are independent of the mass density. Since it is a theoretical value, in the following it is referred to be “physical” or theoretical quantity. It is needed to be underlined that only the “effective” or apparent properties are measurable which may differ from the theoretical ones. As Michalis et al. [59] draw the attention to the “effective” viscosity, the theoretical one must be corrected for rarefied gas flows as a function of the Knudsen number. These corrections are devoted to facilitating the phenomenological descriptions and can be compared to the above-mentioned measurements [59,60,61,62,63].

**Figure 2:**
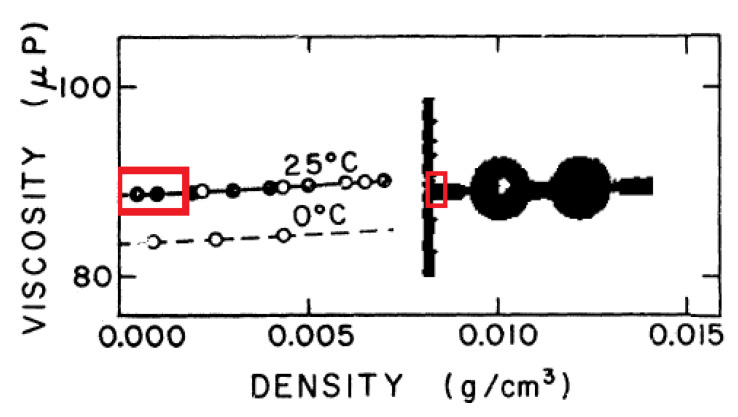
Density dependence of viscosity for dense gases when the non-zero viscosity at zero density appears. The original measurements can be found in [66]. Here, the red boxes show the region of interest together with the extrapolation to zero density.

As the measured effective viscosity shows mass dependence, it requires corrections in both directions: from the rarefied state to the extremely dense states (up to the magnitude of $10^{3}$ MPa). One example is the Enskog-type correction [63,64,65]. It is in good agreement with particular experiments that are devoted to measuring the density dependence of shear viscosity for dense states [64,66,67,68]. These measurements do not aim to investigate low-pressure behavior. Extrapolating the “dense data” to zero, they show the presence of non-zero viscosities at zero density (see Figure 2 for details) [56,60,66,69].

**Figure 3:**
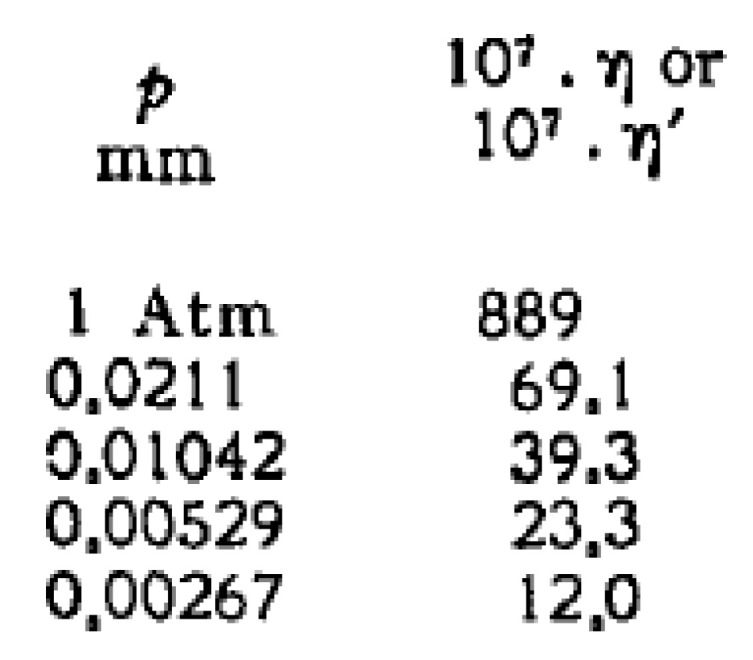
Pressure dependence of viscosity for rarefied gases at room temperature. The original data can be found in [72] which is only partially depicted here.

On the other hand, the measurements of Itterbeek et al. [70,71,72] demonstrate decreasing viscosity by decreasing the pressure. It can be only piecewise linear, its steepness changes drastically at very low pressures (1–10 Pa), and the viscosity tends to zero (Figure 3). However, it is extremely difficult to perform viscosity measurements at such a rarefied state. The outcome of the experiment depends on the size of the apparatus and could influence the results. Furthermore, evaluating such viscosity measurement that corresponds to high Kn number, the classical Navier-Stokes theory may be insufficient and a non-local theory should be used. So to say, there could be some uncertainty regarding the values of the viscosities. From practical point of view, the density dependence seems to be natural and the measurements of Itterbeek et al. also strengthen this expectation: at zero density, there is no viscosity. From a theoretical point of view, this is contradictory to the kinetic theory in the sense of the non-zero value for viscosity in the zero-density limit. Beskok and Karniadakis [73], to overcome that contradiction, suggested a correction that is inversely proportional with the Knudsen number. The correction of the “physical” viscosity is improved by Roohi and Darbandi [74], too.

From a practical point of view again, these effective quantities are essential for modeling, for instance see [59,75,76,77,78,79]. In these papers, generalized constitutive laws are proposed in which apparent quantities play a central role and are used as being the coefficients in the constitutive equations, this is the case also in NET-IV. These referred problems (rheology, non-Newtonian fluids, biological materials) demonstrates that the apparent transport coefficients can depend on various quantities, especially in complex materials. Since the theoretical coefficients differ from the apparent one, at least in the sense of density dependence, it could influence the scaling properties of a model. It is discussed in the following.

For the sake of complete comparison with the work of Arima et al., the same assumptions are used, i.e.,

- both viscosities and the thermal conductivity are constant:
  $\nu =8.82\times 10^{-6}$ Pas, $\eta =326\times 10^{-6}$ Pas and λ=0.182 W/(mK), respectively.
- all relaxation times and the coupling coefficients have 1/ρ dependence.

These follow from a scaling requirement which is discussed in the next section.

### 2.2. Frequency-Pressure Dependence

It is stated frequently in the early experimental papers [51,52,53] that the behavior of a gas depends on the ratio of frequency and pressure alone. From the point of view of kinetic theory, this scaling property is natural and follows from the Boltmann equation.

However, it is not that straightforward from a continuum point of view. Although this scaling is correct based on the experimental data [50], it requires constant transport coefficients, 1/ρ dependence in the new parameters (relaxation times and coupling coefficients) and ideal gas state equation. These assumptions work for the corresponding evaluations; it could not be valid for an extensive pressure (or density, accordingly) domain, e.g., from 10 Pa to $10^{8}$ Pa as the apparent (It is worth to note again that the continuum models consist apparent (or measurable) coefficients in the constitutive equations.) viscosities and the thermal conductivity do depend on the pressure. Moreover, it is not clear how that scaling would appear using a more general equation of state.

Furthermore, it would worth investigating the scaling of relaxation times as the particle-wall interaction starts to dominate the process instead of the particle-particle collisions. In this kind of process, the characteristics of relaxation times are changed and thus changing the scaling properties of the equations. This could be the validity limit of the pure 1/ρ dependence. For example, in the experiments of Meyer and Sessler the lower pressure limit is around 0.2 Pa in which such particle-wall constribution can be especially important to consider.

This scaling property can be easily demonstrated for the classical Navier-Stokes-Fourier model by calculating the dispersion relation using the previous assumptions. Then, there no one will find terms containing the frequency ω and the pressure *p* separately, as follows.

Assuming the common $e^{i(\omega t-kx)}$ plane wave solution of the system (7) with the usual wave number *k* and frequency ω,

(7) $$\begin{array}{cc} \partial_{t}\rho +\rho_{0}\partial_{x}v= & 0,\\ \rho_{0}\partial_{t}v+\partial_{x}\Pi_{d}+\partial_{x}\Pi_{s}+RT_{0}\partial_{x}\rho +R\rho_{0}\partial_{x}T= & 0,\\ \rho_{0}c_{V}\partial_{t}T+\partial_{x}q+R\rho_{0}T_{0}\partial_{x}v= & 0,\\ q+\lambda \partial_{x}T= & 0,\\ \Pi_{d}+\nu \partial_{x}v= & 0,\\ \Pi_{s}+\eta \partial_{x}v= & 0, \end{array}$$

and omitting the detailed derivation, one obtains the following expression for phase velocity $v_{ph}=\frac{\omega }{k}$:

(8) $$\begin{array}{c} v_{ph}^{2}=\frac{cRT\rho^{2}+R^{2}T\rho^{2}+ic\eta \rho \omega +i\lambda \rho \omega +ic\nu \rho \omega }{2c\rho^{2}}+\\ +i\frac{\sqrt{\rho^{2}\left(-4c\lambda \omega \left(-iRT\rho +\left(\eta +\nu \right)\omega \right)+{\left(iR^{2}T\rho -\lambda \omega +c\left(iRT\rho -\left(\eta +\nu \right)\omega \right)\right)}^{2}\right)}}{2c\rho^{2}}. \end{array}$$

Expanding all the terms within Equation (8), the $v_{ph}=v_{ph}\left(\omega /p\right)$ dependence becomes visible and all the experimental data can be evaluated without calculating the pressure (or the mass density) independently from the frequency. In the case of the generalized NSF model, the situation is the same, and the previous assumptions ensure such scaling. Here, the final remark is made from an experimental point of view: the frequency and the pressure are separately controlled and should be documented in this way. Then, in a continuum model, the pressure dependence in any parameter could be implemented without any problem, and the model would be free from assumptions that may be made unconsciously. Moreover, it could extend the validity region of this modeling approach.

### 2.3. Evaluations, Comparisons and Conclusions

**Figure 4:**
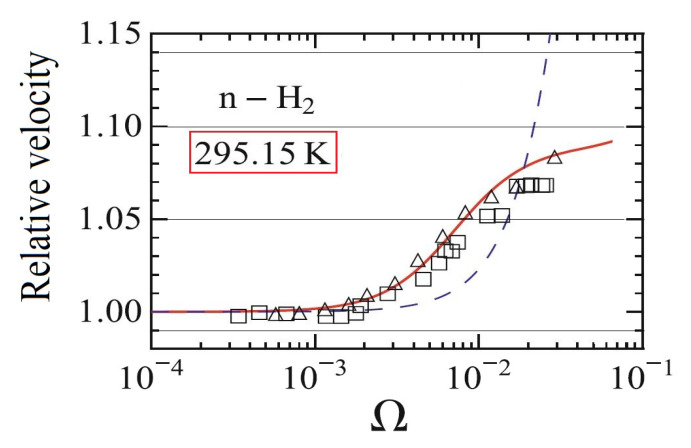
Calculations of Arima et al. [24]. The solid red line shows the prediction, the squares and triangles are referring to different experimental data; here, the triangles represent the data from Rhodes [50]. The dashed line shows the behavior of the Navier-Stokes-Fourier equations.

First, let us consider the results of Arima et al. [24,43], see Figure 4 for details. There are some important remarks on their evaluation method:

1. Dimensionless frequency $\Omega =\tau_{d}\omega$ is used instead of ω/p, moreover, $p\tau_{d}\omega /p=\nu \omega /p$ hence the shear viscosity ν is used to rescale the experimental data. Moreover, the papers [24,43] do not reflect the fact that the frequency was constant. It could be confusing hence it hides that the mass density ρ is the real independent variable in the equations in which all new coefficients (relaxation times and coupling parameters) depend on ρ.
2. Regarding the temperature, 295.15 K is used instead of 296.8 K. It is seemingly a small difference; however, when one attempts to calculate the mass density from the data ω/p, it leads to a different value, i.e., slightly inaccurate the data in [24]. As a consequence, even the speed of sound is slightly inaccurate that used for vertical scale.
3. In [24,43], only certain ratios of relaxation times are fitted. One has to know the pressure in order to determine their values.
4. One single parameter set is used to fit the data from papers [50,53,80] (Figure 4). The relevant data is the following: $\tau_{q}/\tau_{d}=1.46$, $\tau_{s}/\tau_{d}=144$, since the coupling coefficients are also proportional with the relaxation times. It is apparent from Figure 4 that for other measurements, even on the same material, these ratios may be different.

Figure 4 demonstrates that kinetic theory can model the behavior of the gas in the rarefied state.

**Figure 5:**
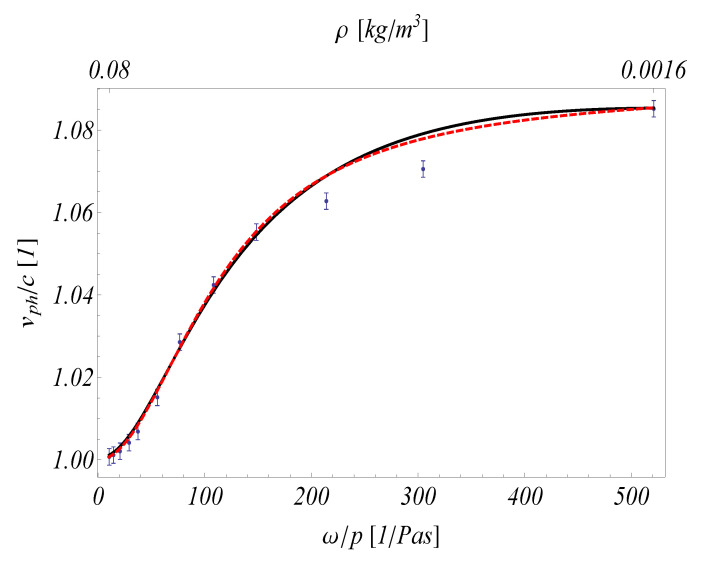
Evaluation using NET-IV (thick black line). The pressure starts at 1 atm and decreases to 2000 Pa, ω=1 MHz. Error bars are placed for each measurement point to indicate the uncertainty of digitalizing data, its magnitude is ±2.5 m/s. The red dashed line shows the results of Arima et al. [24].

Here, using the NET-IV continuum model, only the experiment related to T=296.8 K is considered for demonstrational reasons. It is not intended to evaluate the complete series of measurements. In Figure 5, two horizontal scales are used that intend to indicate the one-to-one correspondence between the ω/p and ρ. It is always possible if the frequency and the temperature are known. Although the fitting procedure is conducted by hand, it is clear that the NET-IV model is also applicable to these problems. However, it is more difficult to do due to more degrees of freedom. Table 1 and Table 2 show the corresponding values of each parameter. For simplicity, in the fitting procedure the ratio of relaxation times was constrained to be the same as for RET, i.e., $\tau_{q}/\tau_{d}=1.46$, $\tau_{s}/\tau_{d}=144$.

## 3. Discussion

The models originated from RET and NET-IV are briefly introduced and tested on a particular experiment performed by Rhodes. It is apparent from the experimental data that the rarefaction in a Hydrogen gas shows substantial deviation from its dense state. Here, the speed of sound data can be evaluated without any obstacles in NET-IV as well, but this evaluation highlights some crucial aspects.

First, we note the difference between the theoretical and measured properties which has great importance in kinetic theory. In contrast, a continuum model does not propose this distinction, it is not possible to calculate the “physical” (theoretical) coefficient without a detailed micromechanism. Moreover, especially from practical point of view, the NET-IV model contains the “effective” (measurable) quantities which have density dependence as well, beside the temperature, according to the referred literature. Since the continuum approach does consist of the measurable transport coefficients, it keeps the continuum model on a more general level that is not restricted specifically, in contrast to the kinetic theory. In the present experimental evaluation, constant viscosities and thermal conductivity are used in order to keep the model compatible with the kinetic theory.

Second, it is advantageous if all the data are given in the experiment; the frequency-pressure ratio could be insufficient, especially when the effective transport coefficients are not constant respect to the pressure. In continuum theory, it does matter what is changed: the frequency, the pressure, or both. In that sense, it is confusing to use dimensionless frequency, especially when all the necessary data are given for the appropriate scale. Besides that fact, the application of dimensionless quantities is still valid, but their use makes it a bit more difficult to interpret the experimental data. Moreover, in the end, even the kinetic approach requires the knowledge about the pressure in order to evaluate the relaxation times and the coupling coefficients.

Moreover, it is also shown that besides the frequency/pressure scaling of experimental data, it is not necessarily obtained in the dispersion relations of a continuum model, merely by restrictive assumptions of constant coefficients. However, it is worth noting that if every experimental data are given appropriately, that is, the frequency and pressure are given separately, then the experimental data become evaluable even if the ω/p scaling is not visible in the dispersion relations. Furthermore, recalling the experimental data of Itterbeek and Gracki et al. in which the viscosity depends on the mass density, this is clear that its inclusion into the models would strongly influence the scaling properties as well. Thus, one question remains: which transport property is important from the aspect of the scaling, the theoretical one or the measurable one?

The difference between the presented approaches originates in the preassumptions. Using kinetic theory, a detailed interaction is assumed, and it restricts some degrees of freedom. On the other hand, in NET-IV, it is not necessary to make any preassumption regarding the type of the fluid, while in kinetic theory it is fixed as a first step, and the derived model such as Equation (5) will be valid only for this restricted case, in the price of the number of free coefficients. As mentioned in the introduction, one of the main difficulties is the fitting of free parameters in the model of NET-IV (4), that is, 7 coefficients are to be fitted instead of 3 that presented by Equation (5). It can be reduced if one adopts some knowledge from the kinetic approach and applies in the continuum model, such as the ratio of relaxation times. This disadvantage could be an advantage if the number of equations is still enough to describe a three-dimensional process on the contrary to the kinetic theory where the momentum expansion generates a large system of equations for a more general situation, for instance in the low temperature heat conduction problems.

As a final remark, it is essential to realize that these approaches extend each other from different directions. While NET-IV keeps the models as general as possible, RET builds a detailed one, with much more knowledge about the coefficients. Although the underlying physical picture is different, the resulting system of equations can be compatible with each other.

## Figures and Tables

**Table 1:** Fitted relaxation time coefficients for continuum model.

$\tau_{q}=\frac{t_{1}}{\rho }$, $t_{1}=\left[\frac{kg\cdot s}{m^{3}}\right]$	$\tau_{d}=\frac{t_{2}}{\rho }$, $t_{2}=\left[\frac{kg\cdot s}{m^{3}}\right]$	$\tau_{s}=\frac{t_{3}}{\rho }$, $t_{3}=\left[\frac{kg\cdot s}{m^{3}}\right]$	$\tau_{q}/\tau_{d}$	$\tau_{s}/\tau_{d}$
$4.526\times 10^{-9}$	$3.1\times 10^{-9}$	$4.464\times 10^{-7}$	1.46	144

**Table 2:** Fitted coupling coefficients for continuum model.

$\alpha_{12}=\frac{a_{12}}{\rho }$, $a_{12}=\left[\frac{kg\cdot s}{m^{3}}\right]$	$\beta_{12}=\frac{b_{12}}{\rho }$, $b_{12}=\left[\frac{kg\cdot s}{m^{3}}\right]$	$\alpha_{21}=\frac{a_{21}}{\rho }$, $a_{21}=\left[\frac{kg}{m\cdot s}\right]$	$\beta_{21}=\frac{b_{21}}{\rho }$, $b_{21}=\left[\frac{kg}{m\cdot s}\right]$
$1.7\times 10^{-6}$	$1.27\times 10^{-6}$	$1.3\times 10^{-4}$	$1.55\times 10^{-5}$
